# Treatment of Permethrin Toxicosis in Cats by Intravenous Lipid Emulsion

**DOI:** 10.3390/toxics10040165

**Published:** 2022-03-30

**Authors:** Simona Di Pietro, Annastella Falcone, Francesca Arfuso, Melissa Pennisi, Giuseppe Piccione, Elisabetta Giudice

**Affiliations:** Department of Veterinary Sciences, University of Messina, 98168 Messina, Italy; dipietros@unime.it (S.D.P.); annastella.falcone@unime.it (A.F.); melissa.pennisi@unime.it (M.P.); gpiccione@unime.it (G.P.); egiudice@unime.it (E.G.)

**Keywords:** antidote, feline, lipid resuscitation, pyrethroids, soybean oil

## Abstract

Background: In this study, the clinical response to treatment with intravenous lipid emulsion (ILE) for nine cats intoxicated with permethrin has been described. Methods: The enrolled cats showed acute onset of seizures, tremors and hypersalivation that were partially controlled with the administration of benzodiazepines and intravenous fluid therapy. Due to worsening clinical signs, intravenous lipid emulsion (intralipid 20%; 1.5 mL/kg in bolus, followed by 15 mL/kg for 1 h in infusion) was administered. Results: After ILE treatment, all cats recovered rapidly and were discharged within 24–54 h. Intravenous lipid emulsion appears to be a relatively safe and inexpensive alternative, with a short hospitalization time. Conclusions: Intravenous lipid emulsion could be an interesting therapeutic option in emergency and critical care protocols for permethrin toxicosis.

## 1. Introduction

Permethrin toxicity has been reported by the Animal Poison Control Center of the American Society for the Prevention of Cruelty to Animals (ASPCA) [1] as one of the most common intoxications in cats, and a high prevalence has also been observed in Italy [2]. Most of these cases are believed to be due to owners mistakenly applying dog products, but exposure to permethrin can also occur after oral ingestion or by grooming with dogs that have been recently treated [3,4]. Pyrethrins are natural substances extracted from botanical species of Chrysanthemum; the term “pyrethroid” refers to similar molecules obtained synthetically, which show an increased photostability compared to the natural forms. Pyrethrins are relatively safe for controlling ectoparasites due to anatomical and physiological differences between mammals and insects. Nevertheless, cats frequently show severe clinical signs after pyrethrin exposure, presenting to the emergency services as unstable patients [5]. The pyrethroids are excreted because of their oxidation or glucuronidation. The reduced ability of glucuronidation in cats can contribute to the accumulation of metabolites and lower efficiency of the detoxification process [5,6]. Pyrethroids act primarily on the central and peripheral nervous system by reversibly altering the functionality of the axon sodium channels, prolonging conduction of the nerve impulse and causing repetitive stress on the nerve fibre [6]. Indeed, these compounds bind to the voltage-gated sodium channels of myelinated nerves, slowing their closure and resulting in repetitive neuronal discharge [6]. This effect is magnified by low temperatures. Because of their lipophilic properties, pyrethrins easily pass through the blood-brain barrier and can trigger clinical signs in the central nervous system. There is evidence of the accumulation of pyrethroids in the central nervous system even when the haematic concentration is low [2]. Clinical signs of permethrin intoxication in cats are characterized by tremors, muscle fasciculations, twitching, hyper-salivation, mydriasis, pyrexia, and, in severe cases, seizures and coma. Other clinical signs include ataxia, tachypnoea, hyperexcitation, and hypothermia. As there is no antidote, conventional treatment protocols aim at controlling the clinical signs and consist of early control of seizures and tremors, decontamination, and supportive care while the toxins are metabolized and excreted. Benzodiazepines are commonly used to control seizures. In rats, methocarbamol has been shown to reduce the clinical neuroexcitatory signs (i.e., muscle tremors) and mortality of pyrethroid toxicoses [3]. Although commonly used [3], the efficacy of methocarbamol for permethrin toxicoses in cats has not been fully evaluated, and it is not available in many countries, including Italy. Supportive care consists of intravenous fluid therapy, body temperature control, and management of eventual complications caused by prolonged seizures, such as hypoglycemia, the onset of non-cardiogenic pulmonary edema and increased intracranial pressure [7]. In recent years, intravenous lipid emulsion (ILE) has been considered a new treatment option in human and veterinary medicine for a variety of intoxications caused by non-lipophilic and lipophilic molecules, including permethrin [7,8,9,10]. In the past decade, numerous reports have been published on the use of ILE to treat dog and cat toxicoses [6,7,8,9]. Despite the growing scientific evidence, knowledge gaps remain in the mechanism of action, optimal concentration, dosing rates, efficacy, and adverse reactions. Therefore, its use in veterinary toxicoses is still considered investigational. Further investigation would be necessary to establish which toxicants best respond to ILE, quantify its efficacy, and record clinical data. Regarding permethrin toxicosis, there are currently some case series and studies in which the use of ILE is reported [3]. An optimal dosing regimen of ILE has not been established. A commonly reported dose of a 20% ILE is a continuous rate infusion of 0.25 mL/kg/min for 30–60 min, preceded or not by a bolus of 1.5 mL/kg [3]. As previously reported, ILE consists of a sterile 10% or 20% fat emulsion manufactured as parenteral nutrition, made up of 10% or 20% soybean oil, egg phospholipids, glycerin, and water [11]. The soybean oil consists of predominantly unsaturated fatty acids: linoleic acid, oleic acid, palmitic acid, α-linolenic acid, and stearic acid [11]. This study describes the use of ILE for the treatment of permethrin toxicosis in nine cats.

## 2. Materials and Methods

The survey is a retrospective study on the medical records of cats visited at a veterinary teaching hospital during the previous year (2020). The enrolment criteria included: direct application of a permethrin product by their owner, reduced or no efficacy to classic treatment (anticonvulsants, fluid therapy), and client consent form by the owner. Nine cats met the inclusion criteria. All cats enrolled in the study were presented to the emergency service for convulsive seizures following the accidental administration of a canine spot-on pesticide approximately 6–13 h prior to presentation. This work involved the use of non-experimental animals only. The cat owners were informed about the methods and purpose of the study and gave their written informed consent. Case 1: A 6-month-old, 2.4 kg, intact male, outdoor domestic European short-haired (DSH) cat was presented after being found by the owner in the garden showing diffuse tremors, which quickly progressed into convulsive seizures. About 6 h earlier, the owner applied about 0.5 mL of 50% permethrin (500 mg/mL)/10% imidacloprid-containing pesticide (Advantix, Bayer S.p.A., Milano, Italy) for large dogs (>40 kg) on the neck. On presentation, the animal showed hyperexcitation, ptyalism, diffuse and continuous tremors, tonic-clonic seizures, tachycardia and tachypnea; the remainder of the clinical signs were normal. Intravenous access (cephalic vein) was obtained while providing flow-by oxygen, and diazepam (1 mg/kg) (Ziapam, Ecuphar Italia s.r.l., Milano, Italy) was administered intravenously (IV) with a consequent decrease in muscle spasms. Obvious twitching in the head (ears and lips) persisted. The cat was washed with mild detergent and warm (35–40 °C) water for dermal decontamination. In addition, intravenous fluid therapy was started using Ringer’s lactate solution (4 mL/kg/h) (Ringer’s lactate solution; Fresenius Kabi, Isola della Scala Verona, Italy). For economic reasons, laboratory tests were limited to determining glucose and PCV/TS. After 1 h, tremors became more severe. The attending clinician decided to administer a lipid emulsion of 20% soybean oil (Intralipid 20, Fresenius Kabi, Isola della Scala Verona, Italy). The first bolus of 1.5 mL/kg was administered over 5 min, followed by a constant rate infusion (CRI) at 0.25 mL/kg/min (15 mL/kg/h) for 60 min. Case 2: A 72-month-old, 4.3 kg, spayed female indoor and outdoor DSH cat was presented for acute onset of tremors, muscle fasciculations and seizures. About 13 hr before presentation to the emergency service, canine Advantix (1 mL; for dogs of 4–10 kg) of the cohabiting dog was applied by mistake on the cat. Clinical signs started about 4 h after administration, quickly evolving into intermittent seizures, which started 1 h before admission. The cat showed hyperexcitation, ptyalism, and generalized muscle fasciculation evolving into a seizure on clinical examination. Diazepam and crystalloids were administered intravenously as described for the previous case, leading to improvement in clinical signs; facial twitching persisted. The cat was bathed, and intravenous fluid therapy was started; the laboratory tests performed were glucose and PCV/TS. After 12 h, generalised muscle fasciculations appeared and rapidly worsened; therefore, intralipid administration was started at the same dosage in the previous case. Cases 3–9: Seven cats belonging to the same colony of DSH cats, aged 6–77 month-old, 3 spayed female, 4 male (2 intact and 2 castrated), with a mean (± SD) body weight of 4.5 ± 0.57 kg, were presented to the emergency service. A few hours before admission, the owner of the colony, following the suggestion of a seller, had applied Advantix for large dogs (>40 Kg) on all the cats of the colony, distributing it empirically (about 0.5–1.0 mL/cat). The owner had difficulty capturing all the animals. Therefore, at first, she was able to bring only three of the cats (cases 3–5), while the remaining ones were presented the next morning, 12 h later (cases 6–9). On clinical examination, all cats showed hyperexcitation, ptyalism, severe generalised tremors, and seizures (cases 3–5). Due to animals’ poor compliance, they were first sedated with butorphanol (0.1 mg/kg intramuscularly, IM) (Dolorex, Msd Animal Health, Milano, Italy). Afterward, an intravenous catheter was applied, and midazolam (0.3 mg/kg) (Midazolam, Ipnovel; Roche, Monza, Italy) and maintenance fluid therapy were administered intravenously, resulting in only partial improvement of the clinical signs. All the cats were partially washed, and a minimum database was performed (glucose, PCV/TS). After 60–90 min, the cats’ tremors became more severe. The attending clinician decided to administer ILE at the same dosage as in the previous cases. This was also due to the owner’s financial difficulties in keeping all animals hospitalized. In case 1, immediately after the bolus, the cat showed a marked response with a visible reduction of clinical signs that completely disappeared 10 min after the CRI was started. At the end of the ILE administration, maintenance fluid therapy with Ringer’s lactate solution continued throughout the next 10 h. About 12 h after the admission, obvious tremors reappeared and quickly progressed as diffuse. After having ruled out frank hyperlipidemia (micro-hematocrit tube), an additional course of ILE treatment (15 mL/kg/h for 60 min) was administered, leading to a positive response with a complete resolution of tremors. Fluid therapy was continued, and the cat was discharged the following day, 40 h after presentation, with close monitoring at home by the owner. In case 2, neurological signs improved rapidly but not completely, with a mild facial twitching persisting after intralipid administration. After 18 h of fluid therapy, muscle fasciculations were again worsening, and intralipid treatment (15 mL/kg/h for 60 min) was repeated, having ruled out obvious hyperlipidemia. After another 24 h of fluid therapy (54 h from hospitalization), the cat was discharged, free from clinical signs. In cases 3–9, the clinical signs improved rapidly and completely during ILE infusion. Fluid therapy was continued during the hospitalization. No neurological signs reappeared, and all the cats were discharged after 24 h of hospitalization.

## 3. Results

In case 1, immediately after the bolus, the cat showed a marked response with a visible reduction of clinical signs that completely disappeared ten minutes after the CRI was started. About 12 h after the ILE admission, tremors reappeared and progressed as diffuse, but after an additional course of ILE treatment, a positive response with complete resolution of tremors was observed.

In case 2, after ILE administration, neurological signs improved, but mild facial twitching persisted. Thus, ILE treatment was repeated, and after 54 h of hospitalization, the cat was discharged, free from clinical signs.

In cases 3–9, the clinical signs improved rapidly and completely during ILE infusion. No neurological signs reappeared, and all the cats were discharged after 24 h of hospitalization.

Table 1 shows clinical and laboratory parameters (minimum database) performed on each cat. Table 2 summarizes the main clinical signs observed at the cats’ admission and their response to treatments and hospitalization times.

## 4. Discussion

Based on the public database of the Animal Poison Control Center of the ASPCA, spot-on products containing permethrin labeled for canine use only can cause serious toxicosis if applied on cats [1]. Products that contain over 40% permethrin are labeled for use in dogs only, many of which can be purchased without veterinary advice. There has been significant concern in the veterinary community about the inadequate labeling of these products and the lack of control over their sale, which has resulted in many deaths of feline patients. In the present case series, toxicosis occurred because of the application of canine products due to the owner’s mistake in the first two cases and the seller’s erroneous advice in colony cats; the actual product amount was undetermined in each case, but was probably higher than 100 mg/kg. Although the minimum toxic dose of permethrin is unknown, life-threatening toxicosis could be caused by a dose lower than 100 mg/kg (1 mL of 45% permethrin if applied dermally on a four to five kg cat) [8]. Moreover, there is no reported correlation between the amount of permethrin applied and the severity of clinical signs induced [3]. No real antidote or antagonist is available, and treatment recommendations include intravenous fluid support to maintain electrolyte balance, hydration to promote diuresis, and dermal decontamination with mild detergent and warm water to prevent further absorption of the toxins. Benzodiazepines, such as diazepam or midazolam, are usually used for seizure control. However, more expensive drugs, such as propofol and alfaxalone, can be used in case of ineffectiveness of benzodiazepines, and mechanical ventilation may be required in the most severe cases. Permethrins are likely to cause tremors. The muscle relaxant drug methocarbamol is usually preferred to control muscle fasciculations and spasms, but at the time of this study, the drug was not available in Italy or other European countries. On average, recovery time from permethrin toxicosis is estimated to be between two or three days, although, in some cases, it can last up to seven days [9]. In the last decade, an increasing amount of evidence supports the use of ILE to reverse or attenuate the clinical manifestations of many lipophilic toxins [9]. Permethrins are very lipophilic compounds with a lipid solubility (log P) of 6.5; therefore, ILE would be expected to be an effective treatment. Scientific evidence focuses on the specific mechanisms underlying lipid resuscitation therapy [12]. The most widely accepted theory states that the effects are caused by the so-called “lipid sink and lipid shuttle”. According to this theory, lipid infusion contributes to removing the toxin from tissues and decreasing its toxic effects [7]. In particular, the expansion of the lipid partition in the blood sequestrates the lipophilic toxins in the intravascular space, separating them from plasma or the target organs (nervous and muscular tissue). The specific dosage for intravenous boluses and the continuous infusion of lipids have not been assessed yet in animals treated for drug toxicosis. The LD50 has only been established in rats as 68 mL/kg [13]. In the current survey, the total amount of ILE administered was lower (16.5–33 mL/kg), in line with previous reports on cats [14]. This volume is also in agreement with the ASPCA Animal Poison Control-recommended dose for dogs (1.5 mL/kg bolus followed by 0.25–0.5 mL/kg/min CRI). In a randomized controlled trial for the treatment of permethrin toxicosis [11], 20 cats were successfully treated, receiving a dosage of 15 mL/kg of 20% ILE over 60 min. The authors decided not to use a bolus like in toxicoses producing life-threatening cardiotoxicity such as with local anaesthetic overdose. All cats in this case series survived to discharge. In all the cases except one, the effect of ILE appeared to cause marked clinical improvement. It might have hastened recovery, probably thanks to its prompt administration when the toxins were still mostly in circulation. Moreover, in the cats of the colony, only one cycle (16.5 mL/kg) of ILE treatment was enough. In case 2, the effects were only transitory, probably due to the delay (25 h) in ILE administration, but it was still effective in controlling neurological signs without the addition of other anticonvulsants. As a matter of fact, one cycle of ILE was able to control the neurotoxic effects of permethrin for about 12 h, thus allowing its metabolization by the organism, which was complete in the case of the colony cats. In the first two cases, the reappearance of neurological signs could be related to inappropriate coat decontamination with a continuous release from the skin or to the metabolization of circulating lipid, no longer sequestering the toxin. Although the dosage of lipemia has not been done, the finding of a clear non-lipemic plasma in the microhematocrit tube would seem to support this last hypothesis. On the other hand, assessing lipemia is strongly recommended to reduce the risk of adverse effects. Side effects of ILE are rare and are mostly due to long-term use in parenteral nutrition. According to previous findings [6,8], none in our case series has shown adverse reactions. In all the treated cats, benzodiazepines (diazepam or midazolam) were initially administered to control neurological signs. Midazolam can be a better option in uncooperative cats for the possibility of IM administration. Furthermore, the colony cats were all previously sedated with butorphanol IM, allowing venous catheterization and decontamination. In all 9 cases, the ILE bolus was administered with a significant reduction in neurological signs and its disappearance about 10 min after the beginning of the continuous infusion. The findings gathered in the current study are in line with a previous study [8], which describes four clinical cases of permethrin poisoning in cats treated with ILE and dexmedetomidine, reporting a more positive outcome in treatment with ILE compared to traditional treatment. In a randomized, controlled clinical study [6], a difference in hospitalization time between the control (saline solution) and ILE-treated cats was not detected, despite the demonstrated earlier improvement in clinical signs of ILE-treated cats. However, the use of methocarbamol may have shortened hospitalization times compared to other published reports. A retrospective study on permethrin feline toxicosis in 42 cases [3] reports that, despite the use of anticonvulsant drugs such as midazolam or propofol used in CRI, the average tremor duration was 35 h. Furthermore, twelve cats were intubated due to respiratory depression caused by propofol. The recorded average hospitalization time was three days [3]. In our study, intubation was not necessary for any of the cats. Among the 9 cats, 7 (the cats of the colony) were discharged within 24 h after presentation, while the other two were discharged within 40 and 54 h, respectively. Our average hospitalization time amounted to 29.11 ± 10.72 h, which is still lower compared to the hospitalization length reported in other case series treated with conventional therapy without methocarbamol [3]. Moreover, all cats were discharged within 30 h of the ILE treatment (24 ± 4.79 h). Finally, but not least, the duration of hospitalization is also dependent on factors other than the clinical status of the animal, including the owner’s economic availability. In cases where methocarbamol is not available, or owner availability is limited, the use of ILE may represent an adjunct life-saving therapy. Based on the results herein found, ILE should be considered an interesting therapeutic option to be included in veterinary emergency and critical care protocols for lipophilic toxin exposure in general and, particularly, in permethrin toxicosis. In all cases, before using ILE, owners must be fully informed of its off-label use and possible adverse reactions. In cases where clinical signs are difficult to control, conventional therapy might not be available, or euthanasia might be considered due to economic concerns, ILE may be considered a relatively safe and inexpensive alternative with a significant reduction of hospitalization time, as it seems to be highly effective in shortening the recovery time for permethrin toxicosis and possibly other fat-soluble toxins. However, prospective randomized controlled trials are required to determine more information about the ideal dosing regimens.

## Figures and Tables

**Table 1:** Clinical (capillary refill time, CRT; rectal temperature, RT; heart rate, HR; respiratory rate, RR) and laboratory parameters of hospitalized cats at first veterinary examination.

Cases				Clinical Parameters				Laboratory Parameters		
	Sex	Age (Months)	Weight (kg)	CRT	RT	HR	RR	Glucose (mmol/L)	Total Solids (g/L)	PCV (%)
1	Intact Male	6	2.4	<2 s	38.5	240	50	5.21	74	45
2	Spayed Female	72	4.3	<2 s	39.1	210	42	6.10	69	42
3	Spayed Female	42	4	<2 s	39.0	190	46	5.43	72	49
4	Spayed Female	30	3.9	<2 s	39.3	205	48	5.99	70	44
5	Spayed Female	64	4.1	<2 s	39.2	200	44	6.10	68	40
6	Intact Male	16	4.8	<2 s	38.6	188	38	6.66	73	38
7	Intact Male	77	4.5	<2 s	38.7	190	42	6.21	69	39
8	Castrated Male	77	5.1	<2 s	38.9	202	48	6.77	68	41
9	Castrated Male	77	5.4	<2 s	38.8	192	46	7.21	71	37
Mean		51.22	4.27		38.90	201.88	44.88	6.18	70.44	41.66
SD		28.30	0.86		0.27	16.22	3.75	0.63	2.18	3.80

**Table 2:** Clinical signs of cats at admission and during the hospitalization, and treatments performed.

	Admission				Hospitalization							
Cases Nr.	Initial Treatment				1st ILE Treatment (Bolus of 1.5 mL/kg in 5 min and 15 mL/kg/h for 60 min)			2nd ILE Treatment (15 mL/kg/h for 60 min)			Discharge (h)	
	Time Post Permetrine Exposure (h)	Clinical Signs	Drug	Clinical Signs Post Treatment	Time Post Initial Treatment (h)	Clinical Signs Pre Treatment	Clinical Signs Post Treatment	Time Post ILE Treatment (h)	Clinical Signs Pre Treatment	Clinical Signs Post Treatment	From Admission	From ILE Treatment
1	6	Tachycardia, tachypnea, ptyalism, hyperexcitation, diffuse and continuous tremors, tonic-clonic seizures, ataxia and mydriasis	D	Ear and lips twitching	1	Diffuse tremors rapidly worsening	None	10	Tremors and ear and facial twitching rapidly worsening	None	40	29
2	13	Hyperexcitation, ptyalism, muscle fasciculations, ear and facial twitching, seizures, and mydriasis	D	Facial twitching	12	Generalized muscle fasciculations rapidly worsening	Mild facial twitching	18	Muscle fasciculations rapidly worsening	None	54	35
3	5	Hyperexcitation, ptyalism, diffuse tremors and tonic-clonic seizures, mydriasis, ataxia	B + M	Tremors	1.5	Severe tremors	None	-	-	-	24	21
4	5	Hyperexcitation, ptyalism, muscle fasciculation and seizures	B + M	Tremors	1	Severe tremors	None	-	-	-	24	22
5	5	Hyperexcitation, ptyalism, seizures, muscle fasciculation and facial twitching	B + M	Facial twitching	1.5	Severe tremors	None	-	-	-	24	21
6	16	Hyperexcitation, ptyalism, and muscle fasciculation	B + M	Tremors	1	Diffuse tremors	None	-	-	-	24	22
7	16	Hyperexcitation, ptialism, and muscle fasciculation	B + M	Tremors	1	Diffuse tremors	None	-	-	-	24	22
8	16	Hyperexcitation, ptyalism, diffuse tremors	B + M	Tremors	1	Diffuse tremors	None	-	-	-	24	22
9	16	Hyperexcitation, ptyalism, and diffuse tremors	B + M	Tremors	1	Diffuse tremors	None	-	-	-	24	22
Mean	10.88				2.57			14			29.11	24
SD	5.44				4.15			5.65			10.72	4.79

## Data Availability

The data presented in this study are available on request from the corresponding author.
